# Osmoregulation and hypoxia tolerance in the cenote isopod *Creaseriella anops*: insights into its distribution in karst subterranean estuaries

**DOI:** 10.1093/conphys/coag009

**Published:** 2026-02-17

**Authors:** Jorge Arturo Vargas-Abúndez, Carlos Rosas, Kurt Paschke, Claudia Caamal-Monsreal, Gabriela Rodríguez Fuentes, Fernando Diaz, Ana Denisse Re Araujo, Maite Mascaró, Efraín M Chávez Solís

**Affiliations:** Unidad Multidisciplinaria de Docencia e Investigación - Sisal, Facultad de Ciencias, Universidad Nacional Autónoma de México, Puerto de abrigo S/N, 97356, Sisal, Hunucmá, Yucatán, México; Unidad Multidisciplinaria de Docencia e Investigación - Sisal, Facultad de Ciencias, Universidad Nacional Autónoma de México, Puerto de abrigo S/N, 97356, Sisal, Hunucmá, Yucatán, México; Instituto de Acuicultura, Universidad Austral de Chile, Los Pinos s/n Balneario Pelluco, Puerto Montt, 5500000, Chile; Centro FONDAP de Investigación en Dinámica de Ecosistemas Marinos de Altas Latitudes (IDEAL), Av. el Bosque 01789, Punta Arenas, 6210445, Chile; Unidad Multidisciplinaria de Docencia e Investigación - Sisal, Facultad de Ciencias, Universidad Nacional Autónoma de México, Puerto de abrigo S/N, 97356, Sisal, Hunucmá, Yucatán, México; Unidad de Química en Sisal, Facultad de Química, Universidad Nacional Autónoma de México, Puerto de abrigo S/N, 97356, Sisal, Hunucmá, Yucatán, México; Departamento de Biotecnología Marina, Centro de Investigación Científica y Educación Superior de Ensenada, Carr. Tijuana-Ensenada 3918, Zona Playitas, 22860, Ensenada, Baja California, México; Departamento de Biotecnología Marina, Centro de Investigación Científica y Educación Superior de Ensenada, Carr. Tijuana-Ensenada 3918, Zona Playitas, 22860, Ensenada, Baja California, México; Unidad Multidisciplinaria de Docencia e Investigación - Sisal, Facultad de Ciencias, Universidad Nacional Autónoma de México, Puerto de abrigo S/N, 97356, Sisal, Hunucmá, Yucatán, México; Unidad Multidisciplinaria de Docencia e Investigación - Sisal, Facultad de Ciencias, Universidad Nacional Autónoma de México, Puerto de abrigo S/N, 97356, Sisal, Hunucmá, Yucatán, México

**Keywords:** Ecophysiology, groundwater, metabolic rates, stygobionts

## Abstract

Groundwater systems of the Yucatan Peninsula form one of the world’s most intricate aquifer systems, supporting a unique and ecologically essential subterranean fauna. The physiological capacities of resident species, and their tolerance or ability to cope with changing environmental challenges is unknown for most species. Considering the vertical stratification of anchialine ecosystems, accelerated climate change and anthropogenic pressures, we sought to investigate the physiological characteristics of a key groundwater-restricted species (stygobionts) that is broadly distributed in the Yucatan Peninsula and has been observed in fresh- and marine groundwater. Thus, we (i) characterized the respiratory metabolism, osmoregulatory capacity and thermal tolerance of the cave isopod *Creaseriella anops* and (ii) evaluated how variations in salinity and oxygen concentration impact its physiological performance and antioxidant defence system. Our findings indicate that *C. anops* is isosmotic at 17.7‰ (580.8 mOsm/kg) and possesses a strong osmoregulatory capacity. When acclimated to freshwater (0‰) at 26 ± 1°C, *C. anops* demonstrated a maximum critical temperature of 33.6 ± 1.3°C and a minimum critical temperature of 19.0 ± 2.0°C, with an aerobic scope of 0.053 mg O₂/g/h. Dissolved oxygen levels (15 to 100% saturation) did not influence routine oxygen consumption rates. Acute shifts in salinity (from 0‰ to 8, 14 or 35‰) initially elevated oxygen consumption rates, which returned to routine levels within three hours across all salinity conditions. This metabolic response was associated with a slight activity increase in antioxidant enzymes and elevated protein carbonylation and lipid peroxidation. In summary, *C. anops* showed outstanding osmoregulatory, metabolic and antioxidant capacities that likely contribute to its wide distribution within the YP’s anchialine ecosystems, providing insights into how this species may respond to future environmental shifts.

## Introduction

The karstic nature of the Yucatan Peninsula (YP), Mexico, allows the marine water to intrude inland, and the precipitation to filter through the rock and float over the marine groundwater (MG), giving place to one of the largest karst subterranean estuaries (KSE; a.k.a., anchialine systems). Such KSE are vertically stratified by abrupt salinity changes (haloclines), which are accompanied by changes in pH, dissolved oxygen (DO) and temperature (Brankovits et al., 2022). Such environmental settings define habitat availability and determine the distribution of stygobionts (Chávez-Solís et al., 2020; Mrozińska et al., 2021; Chávez Solís et al., 2024). Stygobionts are species with an obligate dependence on aquatic subterranean habitats, where they complete their entire life cycle (Gibert et al., 1994; Sket, 2008; Culver and Pipan, 2009; Trajano, 2012), Groundwater organisms exhibit a range of morphological, behavioural and physiological adaptations that allow them to thrive in such oligotrophic and dark environments (Hervant and Malard, 1999, 2012; Sket, 1996). Although this habitat is mostly stable throughout the year (Mejía-Ortíz et al., 2022), rapid changes in oxygen saturation, methane concentrations and vertical shifts of haloclines have been observed with extreme meteorological events such as tropical storms and hurricanes (Kovacs et al., 2017; Brankovits et al., 2022), which are common around the winter-storms season in the YP. Such extreme events inflict physiological stress on stygobionts and may further restrict their habitat availability or decimate populations (Calderón Gutiérrez et al., 2023).

Osmoregulation is a fundamental physiological process by which organisms regulate the concentration of water and solutes within their bodies, maintaining a stable internal osmotic environment despite changes in external conditions (Rahi et al., 2018). Freshwater and many marine organisms allocate a significant portion of their energy budget (5–30% of basal metabolic rate) for maintaining water and ion balance (Sokolova et al., 2012). This is achieved by actively excreting water and counter-gradient ion transport via a suite of ion transporters and supporting enzymes, such as Na^+^/K ^+^ -ATPase and carbonic anhydrase for pH control (Garçon et al., 2021).

Crustaceans exhibit varying osmotic capabilities and mechanisms influenced by their evolutionary and life history traits (McNamara and Faria, 2012; Mcnamara and Freire, 2022), in fact, even closely related taxa may exhibit differing responses to salinity (McNamara and Faria, 2012; Havird et al., 2019; Ballou et al., 2022; Chávez Solís et al., 2024). While most marine species are isosmotic and iso-ionic with seawater, freshwater species typically possess robust osmoregulatory capacities, as they actively adjust internal salt and ion concentrations to differ from those in the surrounding freshwater environment (Garçon et al., 2021). Consequently, organisms inhabiting dynamic environments like estuaries and intertidal zones tend to possess physiological and structural adaptations for both hyper- and hypo-regulation (Havird et al., 2014a; Mcnamara and Freire, 2022). Osmoregulation, as any active physiological response, requires energy input. Therefore, an increase in energetic demand will require a metabolic shift, which produces a greater amount of ROS. Such an increase in ROS triggers the antioxidant system, which uses enzymes to prevent or mitigate cellular damage, in a balancing process around cellular homeostasis.

In addition to salinity, oxygen is another key environmental factor determining distribution of many aquatic species. Oxygen is the last electron acceptor in the aerobic respiration pathway, and thus, it is paramount for the survival of metazoans. Dissolved oxygen (DO) in the YP aquifer is generally far from saturation, spanning from anoxia to approximately 50% saturation (Brankovits et al., 2017, 2022; Ballou et al., 2022). The oxygen availability in groundwater is determined by the rate of oxygen transport from the surface environment and the rate of oxygen consumption in the subsurface (Hervant and Malard, 1999). Additionally, small-scale spatial heterogeneity in DO reflects changes in sediment composition and structure, subsurface water flow velocity, the strength of hydrological exchanges with the surface environment, dissolved and particulate organic matter content and the activity of microorganisms (Hervant and Malard, 1999; Bauer-Gottwein et al., 2011; Brankovits et al., 2022). This implies that organisms in the YP must thrive in habitats with a generally low DO availability while also experiencing variable oxygen concentrations as they move through a mosaic of patches with contrasting DO concentrations (Hervant and Malard, 1999). As a result, stygobionts often exhibit extremely low metabolic rates (MR) compared to their surface-dwelling relatives, although some show an intriguing diversity of metabolic rate patterns (Di Lorenzo et al., 2024). As groundwater is the only freshwater resource in the region, it is subject to anthropogenic pressures such as over-extraction and pollution. The over-extraction of fresh groundwater leads to the salinization of the aquifer by the elevation of MG, which in coastal areas impacts water quality along with the composition of subterranean communities (Shapouri et al., 2016; Velasco et al., 2019). These anthropogenic impacts add to the suite of stressors that stygobionts are required to cope with. Despite the central importance of stygofauna in nutrient and carbon cycles, and thus maintaining aquifer quality, knowledge of their tolerance to increasing environmental changes remains limited (Chávez-Solís et al., 2022).

Of the seven species of stygobiont isopods in the YP, *C. anops* (Creaser, 1936) is one of the most abundant (Ruiz-Cancino et al., 2013; Durán and Álvarez, 2021) and widely distributed in its groundwater systems (Alvarez et al., 2015; Angyal et al., 2020; Durán and Álvarez, 2021). Additionally, it plays a central role in nutrient recycling, as it is a benthic and burrowing species. Although *C. anops* primarily resides in the freshwater layer of these caves, it has been sporadically observed in the halocline and, exceptionally, in the saltwater layer (Benítez et al., 2019). Such observations suggest this species has the capacity to cross the haloclines, yet the metabolic costs of such behaviour and their capacity to remain in both fresh groundwater and MG are unknown.

This study aimed to investigate the physiological capacities of *C. anops* to understand its response to salinity changes and DO reduction, as one of the key species in the Yucatán Peninsula’s KSE. We characterized respiratory metabolism, osmoregulatory capacity and thermal biology, and evaluated the effect of salinity and oxygen concentration on its physiological performance and antioxidant system. By unravelling the possible mechanisms involved in these responses, we provide insights into how this species thrives in such a challenging environment, forecasting its sensitivity to environmental changes.

## Materials and Methods

A total of 203 individuals were collected from the Xtabay cenote (20.499183° N, 87.260842° W), which is part of the Ponderosa System in Quintana Roo, Mexico. Salinity, DO, temperature and pH were previously obtained with a Hydrolab DataSonde5 (see Fig. 1 in Chávez-Solís et al., 2020), and salinity was confirmed onsite with an optical refractometer (Atago, USA, accuracy ±0.5%). Organisms were collected using 1-L plastic bottle traps baited with canned tuna fish. Three traps were laid by SCUBA cave divers in freshwater at the cavern area at depths of 6, 8 and 9 m, from 6 PM to 8 AM the next day. From the total 203 individuals, sixty-one were transferred to an aerated 20-L bottle with water from the cenote surface and stored until *in situ* trials were performed, while the 142 remaining ones were then taken to the laboratory in Sisal, Yucatan.

**Figure 1:**
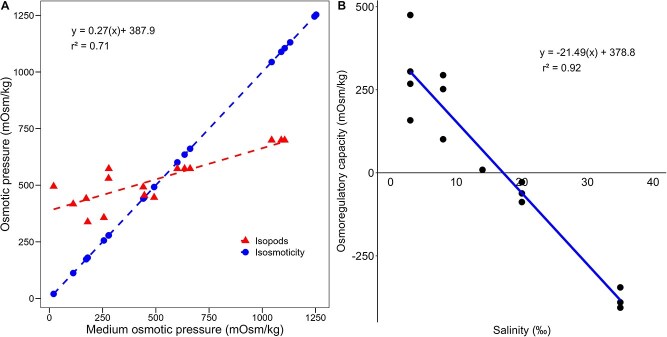
Osmoregulatory response of adult *C. anops* exposed to varying salinities. (**A**) Relationship between internal (organism) and external osmotic pressures. The red regression line deviates from the isosmotic line (blue line, slope = 1), indicating active osmoregulation. (**B**) Osmoregulatory capacity, defined as the difference between internal and external osmotic pressures. Positive values denote hyper-regulation in dilute media; negative values indicate hypo-regulation in saline conditions. Points represent independent replicate values

### *In situ* measurements and experiments

Immediately after collection, 15 adult isopods were transferred from their natural salinity (0‰) into 50-ml plastic vials containing one of the five following target salinities where they were maintained during 8 minutes of exposure: 0, 8, 14, 20 and 35‰ (3 isopods per salinity group). The 8-minute exposure duration was selected to mirror the short-term salinity transitions observed in the field, where *C. anops* sporadically enters the halocline or marine layer for minutes at a time (Benítez et al., 2019; Chávez-Solís, pers. obs.). To prepare the saline solutions, MG (35‰) and fresh groundwater (0‰) were obtained, respectively, from the lower and upper halocline layers of the Ponderosa System. These were then mixed to achieve the desired salinity concentrations, measured with an optical refractometer (±1‰). After the exposure period, the isopods were dried on absorbent paper, and 20 μl samples of hemolymph were collected from each isopod using a hypodermic glass needle inserted into the ventral sinus. Additionally, we measured the osmotic pressure from three to six water samples of each salinity treatment. All osmotic measurements were obtained using a micro-osmometer 3 MO Plus (Advanced Instruments, Inc., Norwood, Massachusetts, USA).

A similar 8-minute salinity exposure was performed for an additional 46 isopods, with 12, 9, 9, 7 and 9 randomly assigned to 0, 8, 14, 20 and 35‰ salinities, respectively. After exposure, the isopods were blotted briefly on filter paper, weighed (OHAUS Explorer analytical balance; NJ, USA; precision 0.1 mg), and immediately snap-frozen in liquid nitrogen. Specimens were then stored at −80°C for subsequent bioindicator analyses.

### Laboratory maintenance

A total of 142 isopods were rinsed with freshwater after collection and transferred to the applied physiology laboratory at the National Autonomous University of Mexico (UNAM) in Sisal, Yucatán, Mexico. Animals were acclimatized to laboratory conditions in a maintenance tank for two weeks in a 250-L flow-through indoor tank with gentle aeration. The tank conditions were maintained at 25.1 ± 1°C, salinity 1 ± 1‰ and pH 8.1 ± 0.4. To provide a suitable habitat, plastic mesh was added to the tank as an artificial substrate, similar to the amphipod aquaculture setup (Vargas-Abúndez et al., 2021). The isopods were fed daily with commercial shrimp feed (Camaronina 35 Purina, Sonora, Mexico), which contains 350 g kg^−1^ crude protein, 80 g kg^−1^ lipids, less than 100 g kg^−1^ ash, less than 50 g kg^−1^ fibre and an energy content of 21.6 kJ g^−1^.

### Critical thermal maximum and minimum

To determine critical thermal maximum (CT max), we randomly selected ten adult isopods from the maintenance tank after acclimation to lab conditions. Each isopod was transferred to a 50-ml beaker containing 20 ml of water from the acclimation tank. These beakers were then placed inside a 40-L aquarium functioning as a thermoregulated bath. Temperature within the beaker was increased at a rate of 1°C/min starting at the acclimation temperature (26 ± 1°C) until animals showed loss of equilibrium or muscular spasm (Ern et al., 2023). While loss of equilibrium could be subtle, it was invariably followed by distinct muscular spasms, which served as a clear and reliable indicator of thermal limits, consistent with standardized protocols for aquatic ectotherms (Lutterschmidt and Hutchison, 1997; Terblanche et al., 2011). Temperature was monitored using a calibrated CheckTemp®1 digital thermometer (Hanna Instruments) (accuracy ±0.1°C) within the beakers, with water temperature serving as a proxy for internal body temperature. A 1000-W immersion heater, coupled with an aeration stone, was used to regulate and elevate the temperature.

For determining critical thermal minimum (CT min), an additional 10 adult isopods were randomly selected from the maintenance tank. Each isopod was transferred to a 50-ml beaker containing 20 ml of water from the acclimation tank. These beakers were then positioned inside a 40-L polystyrene box. Minced ice was carefully placed around the beaker, causing the temperature to decrease at a rate of 1°C per minute, starting from the acclimation temperature. Temperature was also monitored from within each beaker with a CheckTemp®1 digital thermometer (Hanna Instruments). The same behavioural endpoints (loss of equilibrium or muscular spasm) were recorded to determine CT min (Lutterschmidt and Hutchison, 1997; Martínez-Porchas and Hernández-Rodríguez, 2010; Cumillaf et al., 2016).

### Baseline routine oxygen consumption rates and the influence of salinity

The routine oxygen consumption rates of adult *C. anops* were measured individually using intermittent respirometry in 17-ml glass chambers (Svendsen et al., 2016). Thirty-five randomly selected isopods from our maintenance tank (0‰ salinity and 100% oxygen saturation) were isolated and fasted for 24 hours before being placed individually in the glass respirometry chambers. These chambers were filled with water at the acclimation temperature and one of five different salinities: 0, 8, 14, 20 and 35‰. Chambers without isopods were used as control to account for bacterial oxygen depletion.

Intermittent respirometry was performed using an aquarium pump that supplied oxygen-saturated water to each chamber through a hose system attached to each lid. Ten respirometric cycles were performed, with each cycle consisting of 30 minutes of measurement (functionally closed chamber) followed by 30 minutes of water flushing, consisting of ten cycles in total (10 hours in total). To prevent the isopods from becoming hypoxic and stressed during the respirometry trials, the cycles were oxygen replenished when oxygen concentration was reduced by approximately 1 mg/L of oxygen, corresponding to approximately 20% of the chamber’s initial oxygen content.

Oxygen consumption in each chamber was measured by recording the DO concentration at a rate of 1 Hz using flow-through oxygen sensors (Loligo Systems, Denmark) connected via optical fibre to Witrox 4 amplifiers (Loligo Systems, Denmark). Prior to the measurements, each sensor was calibrated with saturated freshwater (for 100% DO) and a 5% sodium sulfite solution (for 0% DO). To reduce stress, trials were conducted in an isolated, semi-dark room and left undisturbed during trials.

Oxygen consumption rate (mg O_2_/g/h) for each cycle was estimated using the following equation:


(1) \begin{equation*} {VO}_2=\frac{{\left[{O}_2\right]}_i-{\left[{O}_2\right]}_f}{\varDelta T}\times \frac{Vr}{ww} \end{equation*}

where [O_2_]_i_ and [O_2_]_f_ are the oxygen concentration (mg L^−1^) at the beginning and the end of the respirometry trial, respectively, ΔT the period of the trial, Vr the volume of water (L) in the respirometry chamber minus the weight (g) of the animal (assuming its density was equal to that of water), and ww the wet weight of the animal (g). Consequently, the consumption rates are expressed as mg O_2_ per g of wet weight every hour. The three lowest consecutive values were selected, averaged and determined as the routine oxygen consumption rate, which in this case is a proxy of standard metabolic rate.

### Effect of isopod size on oxygen consumption

To evaluate the effect of body size on oxygen consumption rates (i.e. metabolic scaling), we supplemented data from the previously described oxygen consumption experiment (animals measured at 0‰ salinity) with measurements from an additional eight isopods, yielding a total of 13 individuals (all measured at 0‰ salinity). For consistency with other studies on stygobitic crustaceans, we estimated dry weight from wet weight using the same conversion factor (15% of wet mass) employed in a recent meta-analysis of metabolic scaling in stygobitic crustaceans (Di Lorenzo et al., 2024).

Metabolic scaling was characterized using the power function *Y* = *aM*^*b* (e.g. Kleiber, 1932), where *Y* is metabolic rate (μg O₂/ind/h), *M* is body mass (dry weight in mg), and *a* (intercept) and *b* (slope) are constants. We applied this equation to natural logarithm-transformed data, such that: *b* > 1 indicates metabolic rate increases faster than body mass; *b* < 1 indicates metabolic rate increases more slowly than body mass; *b* = 1 denotes isometric scaling (metabolic rate increases proportionally to body mass); *b* = 0 implies metabolic rate is independent of body mass.

### High metabolic rate, LMR and thermal metabolic scope

For each salinity group, the thermal metabolic scope (TMS) of an additional ten randomly selected isopods was determined using respirometry, following the methods described by Paschke et al. (2018). This method uses temperature to induce minimum and maximum metabolic rates (temperature-induced metabolic rate; TIMR method), employing the 90th quantile of the critical temperature (CT) for both low and high temperature exposures. Based on the CT data observed in this study (CT max: 33.6 ± 1.3°C and CT min: 19.0 ± 2.0°C), TIMR max was measured at 30°C and TIMR min at 21°C. Closed respirometry was used to determine TIMR max and TIMR min, with a similar chamber and system setup to that used for measuring routine oxygen consumption. TMS was calculated as the difference between the mean values obtained for TIMR max and TIMR min.

### Tolerance to hypoxia

Twenty-four adult isopods were randomly selected from the maintenance tank and transferred to respirometry chambers filled with water at one of four different salinities: 0, 8, 14 and 35‰ (6 isopods per salinity group). The DO concentration was gradually decreased from 100 to 15% saturation at a rate of 14–15% per hour. To achieve this, nitrogen gas was injected through an aeration stone directly into the water of a reservoir tank that supplied water to the respirometry chambers by means of a water pump. A flow meter connected to the nitrogen generator regulated the nitrogen flow, requiring a nitrogen pressure of 15 Kpa to reduce the DO concentration to 15%. Oxygen consumption rates (mg O_2_/g/h) for each cycle were estimated as described for the baseline routine oxygen consumption.

### Assessment of the antioxidant system

The antioxidant capacity of isopods exposed to acute salinity changes for 8 minutes was assessed using various enzymatic assays. Samples stored at −80°C were processed immersed in ice with a Potter–Elvehjem and a PTFE pistil homogenizer, adding Tris buffer (pH 7.4; 0.05 M) to obtain a proportion of 100 mg of tissue per ml of buffer, as described in Rodríguez-Fuentes et al. (2008). The resulting homogenates were individually centrifuged at 4°C and 10 000 g for 5 minutes. The supernatant was extracted and subdivided for measuring superoxide dismutase (SOD), Glutathione-S transferase (GST), acetylcholinesterase (AChE) and carboxyl esterase (CbE). All assays were done in duplicate subsamples. SOD activity and GSH were evaluated using Sigma-Aldrich assay kits 19 160 and CS0260, respectively, by following the manufacturer’s instructions. As described by Góth (1991), with the modifications by Hadwan and Abed (2016), catalase (CAT) activity was measured as the rate of peroxide (H_2_O_2_) reduction after reaction with ammonium molybdate, determined with a spectrophotometer at 374 nm.

To evaluate cellular damage from reactive oxygen species (ROS), lipid peroxidation (LPO) and carbonyl groups in oxidized proteins (PO) were measured in the sampled tissues. LPO was assessed following the manufacturers’ instructions using Peroxi Detect Kit (PD1, Sigma-Aldrich, USA). PO was assessed following protocols developed by Mesquita et al. (2014).

AChE activity was measured using methods in Ellman et al. (1961) adapted to a microplate reader, as described in Rodríguez-Fuentes et al. (2008). CbE activity was measured following the procedures in Hosokawa and Satoh (2002). Some biomarkers require the amount of total protein per volume of sample, which was assessed following methods in Bradford (Bradford, 1976). LPO, PO and GSH were expressed as nmol/mg of tissue, SOD as enzime activiy (U) per mg of protein. AChE, CbE, CAT and GST were expressed as nmol/min/mg of protein.

### Statistical analysis

A linear regression using ordinary least squares was adjusted to the observed internal osmotic pressure of *C. anops* as a function of the external osmotic pressure to determine whether isopods behaved as osmoconformers (i.e. organisms that keep internal fluids isotonic to the environment). If the predicted regression line intersected that of isosmoticity (*b* ≠ 1), there would be evidence of osmoregulation in *C. anops*.

CT max and CT min were measured in native conditions (freshwater) to define the thermal tolerance range (CT_max_—CT_min_), with values reported as mean ± SD. Similarly, TIMR max and TIMR min were quantified to assess the scope for thermal tolerance in freshwater. The relationship between dry weight and oxygen consumption rate was modelled using a linear regression on log-transformed oxygen consumption and dry weight values.

Changes in routine oxygen consumption rates among salinity exposure groups were assessed using a one-way analysis of variance (ANOVA). All parametric tests (*t*-tests, ANOVA, linear regression) were validated for normality and homogeneity of variance using Shapiro–Wilk tests (*P* > 0.05) on the model residuals and visual inspection of residual plots. The relationship between oxygen consumption and oxygen saturation across salinities are presented descriptively, with no statistical test. This was because, at low oxygen saturations, the oxygen consumption rates in experimental chambers converged on the background rates recorded in control chambers (without isopods). The resulting signal-to-noise ratio was too low to distinguish biological consumption from background microbial or instrumental drift, making the identification of a clear oxygen critical point (Pcrit) unfeasible. All univariate tests and data processing were performed using R statistical software (v4.4.1, R Core Team, 2023), and graphics generated using ggplot2 v3.5.1 (Wickham, 2016).

The enzymatic activities and markers of oxidative damage were analyzed by principal coordinate analyses (PCO), followed by permutational multivariate analysis of variance (PERMANOVA; Anderson, 2001) using PRIMER v7 (Clarke and Gorley, 2015). For PCO, a resemblance matrix with dissimilarity measures (Euclidean distance) between each pair of samples was constructed. Raw data were pre-treated with a natural logarithm transformation (Log[*X* + 1]) and *z*-normalization. To assess the statistical significance of the salinity groups identified by PCO, a one-way PERMANOVA was conducted, treating salinity as a fixed explanatory factor. Unrestricted permutation of raw data (999 permutations) was used to generate empirical distributions of pseudo-*F* values under the null hypothesis (Anderson, 2017). *Post hoc* comparisons were applied similarly when the main test indicated significant differences (*P* < 0.05) between at least two centroids.

## Results

### Osmoregulatory capacity

The slope of the isopod’s internal osmotic pressure significantly differed (*t*_14_ = −15.27, *P* < 0.001) from that of the external osmotic pressure, supporting *C. anops* as an osmoregulator (Fig. 1A). The isosmotic point was found at 580.3 mOSm/kg (intersection of regression lines in Fig. 1A), which corresponded to a salinity of 17.7‰ (Fig. 1B).

### CT max and CT min

**Figure 2:**
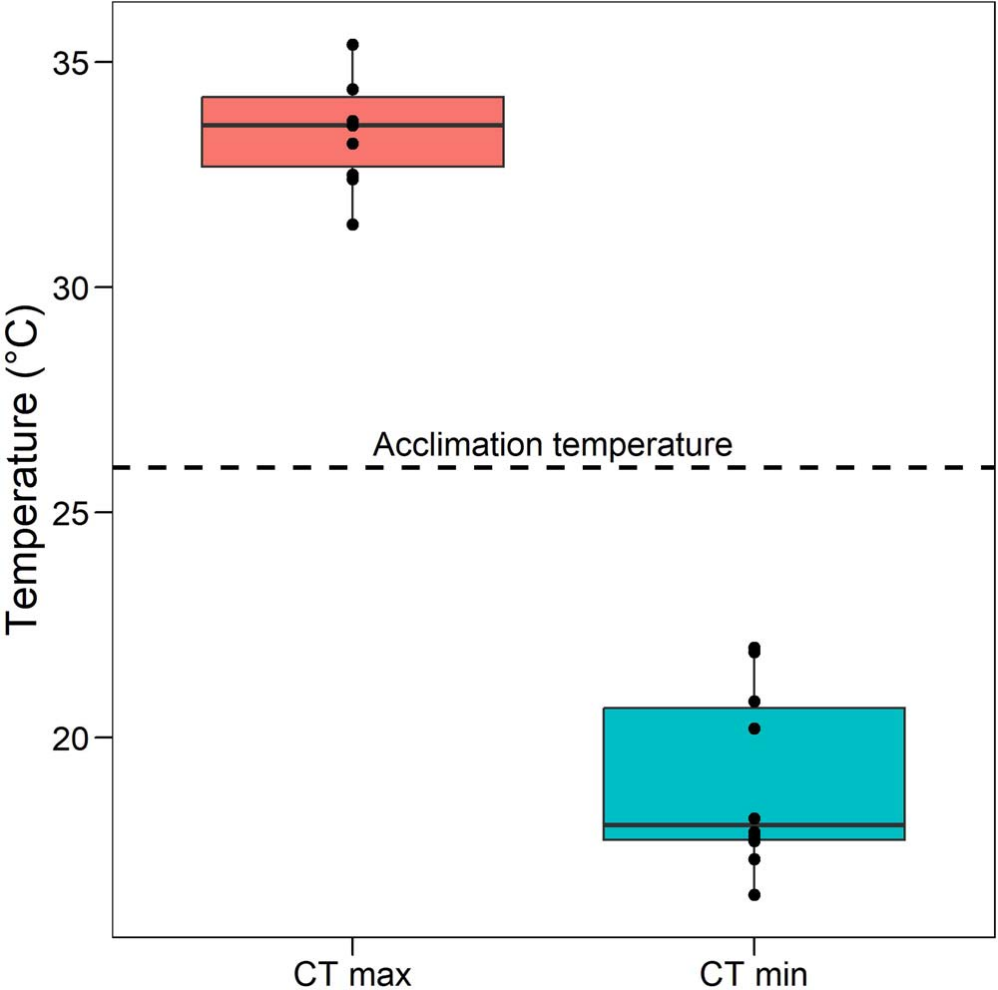
CT max and CT min of adult *C. anops* acclimated to 26°C. Box and whisker shows the median, 25 and 75% interquartile limits and minimum and maximum values. The box represents the IQR, spanning the 25^th^ percentile (bottom edge of the box) to the 75^th^ percentile (top edge of the box), with the horizontal line within the box showing the median (50^th^ percentile). The whiskers extend to the smallest and largest values within 1.5 times the IQR from the first and third quartiles, respectively, while values beyond this range are considered outliers. Individual points represent replicate values

At an acclimation temperature of 26°C, *C. anops* adults showed a mean CT max and CT min of 33.6 ± 1.3 and 19.0 ± 2.0°C, respectively (Fig. 2). Thus, the scope for thermal tolerance was 14.6°C.

### Baseline oxygen consumption rates

**Figure 3:**
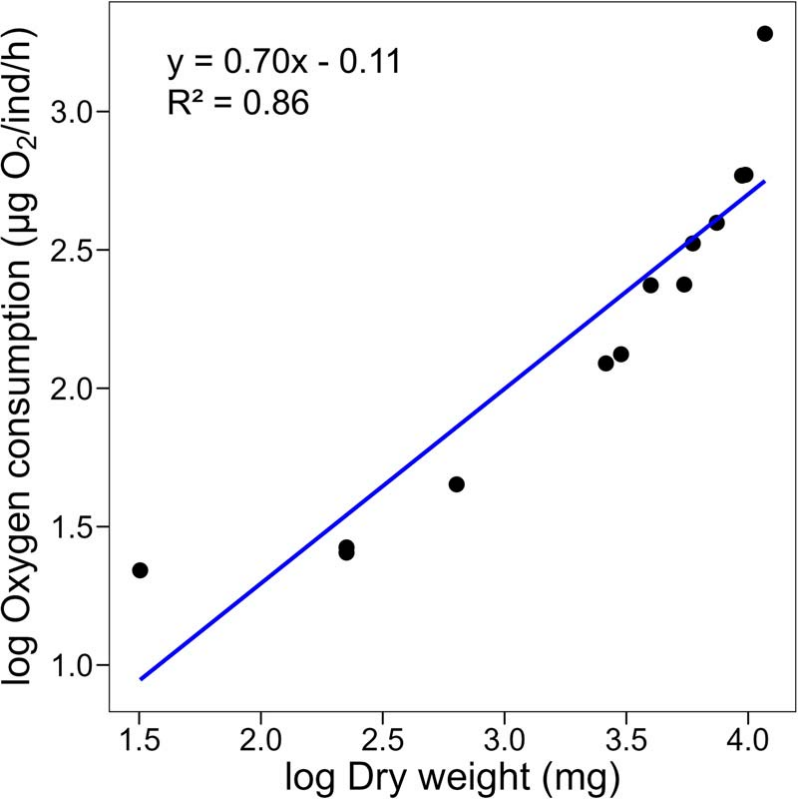
Relationship between individual oxygen consumption rate (μg O₂/individual*h) and dry weight (mg) for *C. anops*, both plotted on a natural logarithmic scale to illustrate metabolic scaling (*b* = 0.70). Individuals were acclimated to 26°C and in their native salinity (0 ± 1‰) Individual points represent replicate values

**Figure 4:**
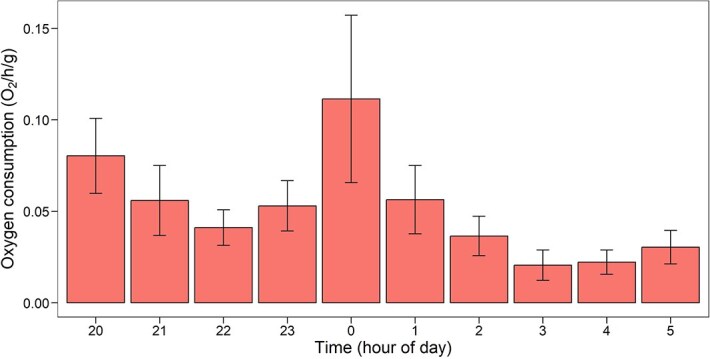
Mean oxygen consumption rate (mg 0_2_/h/g ± standard error) of adult *C. anops* (*n* = 6) acclimated to 26°C and 0‰ salinity throughout the day

In animals acclimated to a salinity of 0‰ and 26°C, the mass (estimated dry weight) had a significant effect on the oxygen consumption rates of *C. anops* (*t*_11_ = 8.10, *P* < 0.001), with a scaling coefficient of 0.70 (Fig. 3). In adult specimens (weighing 265 ± 55 mg), the mean routine metabolic rates at this acclimation temperature was 0.024 ± 0.01 mg O_2_ (g*h)^−1^. Variations in oxygen consumption rates over time were also observed, with two consumption peaks: one associated with the beginning of the respirometry trial (20 h) and another occurring at midnight (0 h.) (Fig. 4). The differences in mean oxygen consumption rates, however, were not statistically significant (*F*_9,5_ = 2.04, *P* > 0.05).

**Figure 5:**
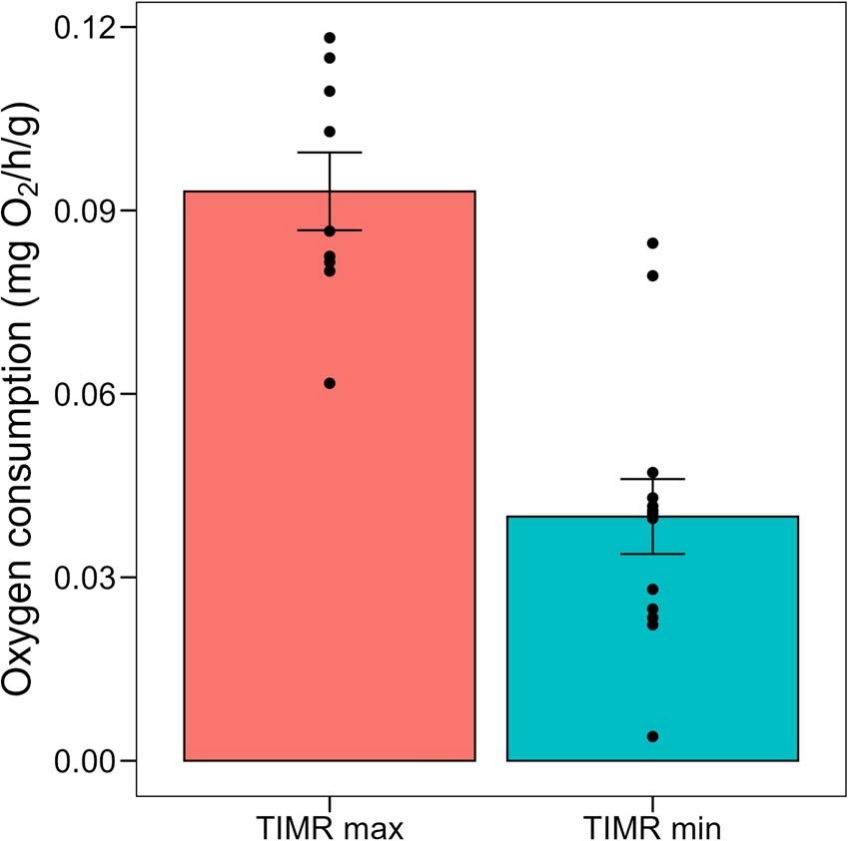
Maximum (TIMR max, measured at 30°C) and minimum temperature-induced metabolic rate (TIMR min, measured at 21°C) of adult *C. anops* acclimated to 26°C. Values are mean ± standard error. Points represent replicate values

Temperature-induced metabolic rates, TIMR max and TIMR min, were 0.093 ± 0.02 mg O_2_/g/h and 0.040 ± 0.02 mg O_2_/g/h, respectively (Fig. 5). Thus, animals showed a thermal metabolic scope of 0.053 O_2_/g/h.

### Effect of acute salinity change on routine oxygen consumption rates

**Figure 6:**
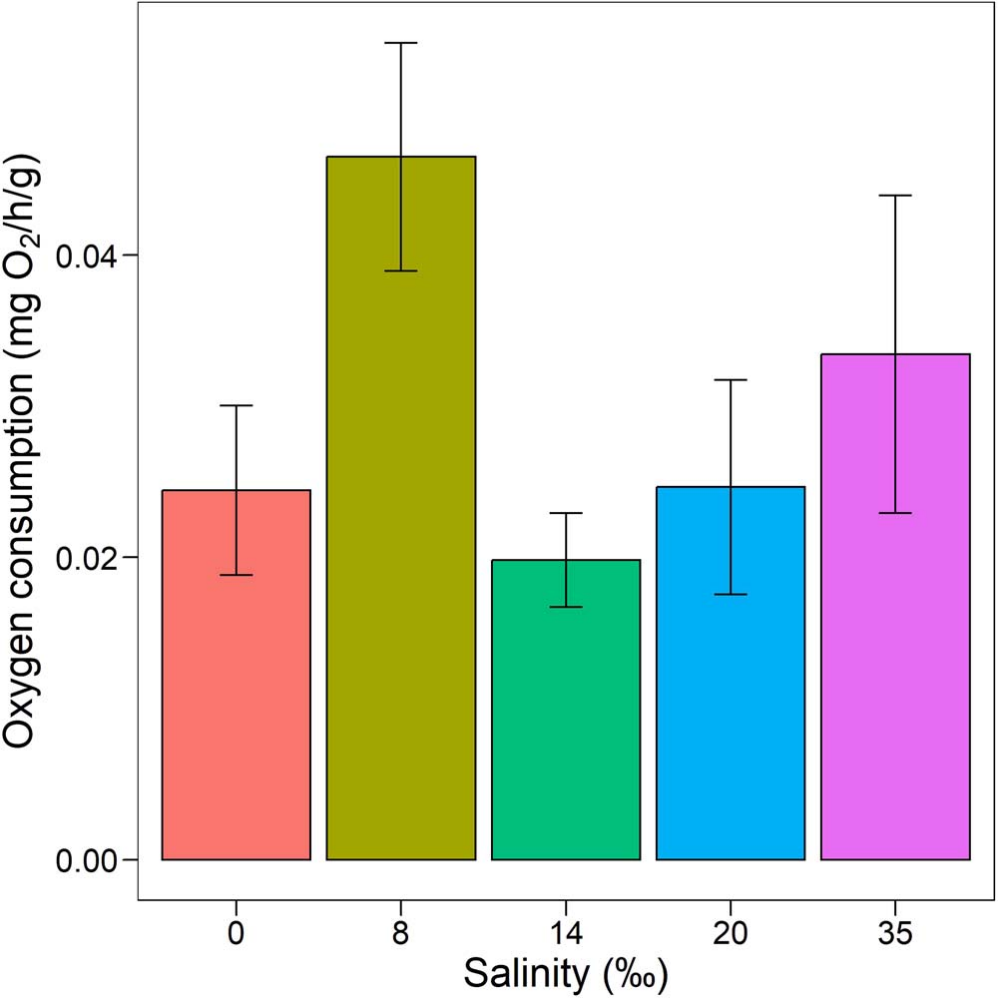
Mean routine oxygen consumption rate (mg O_2_/h/g ± standard error) of adult *C. anops* exposed to different salinities. Replicate values per salinity treatment: *n* = 6–7

Acute salinity change, during respirometry trials, did not significantly affect the routine oxygen consumption rate (*F*_4,5–6_ = 2.04, *P* = 0.12) (Fig. 6). However, it is important to stress that animals measured at 8‰ salinity increased a 2-fold mean oxygen consumption rate (0.46 ± 0.02 mg O_2_/g/h) with respect to 0, 14 and 20‰ groups (0.024 ± 0.01, 0.020 ± 0.01 and 0.025 ± 0.02 mg O_2_/g/h, respectively).

### Tolerance to hypoxia

**Figure 7:**
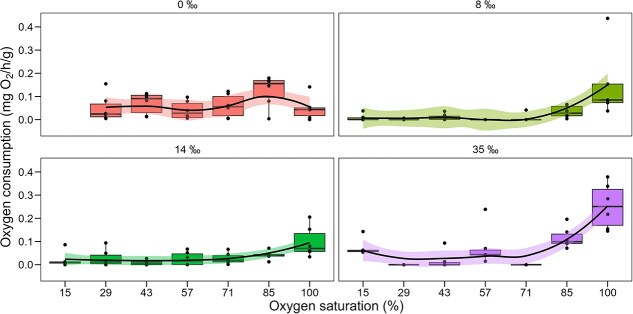
Effect of oxygen saturation (%) on oxygen consumption (mg O₂/h/g) in *C. anop*s isopods acclimated to 0‰ salinity and exposed to acute changes in salinity (8, 14 and 35‰). Boxplots display the IQR, with the box spanning from the 25^th^ to the 75^th^ percentile and the horizontal line within the box representing the median (50th percentile). Whiskers extend to the smallest and largest values within 1.5 times the IQR from the first and third quartiles, while values beyond this range are plotted as outliers. Individual points represent replicate values. To aid interpretation, a trendline with its associated standard error (shaded area, generated by a loess function) is included in each plot

Oxygen consumption rates in *C. anops* were independent of oxygen saturation across all salinity treatments (0, 8, 14 and 35‰), and no *P*crit could be determined within the measured range (Fig. 7). However, exposure to salinity increased metabolic rates at 100% oxygen saturation. Compared to the freshwater control (0‰), oxygen consumption rates were approximately two to three times higher in salinity-exposed groups. The most pronounced increases were observed at 8 and 35‰ salinities, which showed 3-fold and 2-fold rises, respectively.

### Effect of acute salinity exposition on the antioxidant system

**Figure 8:**
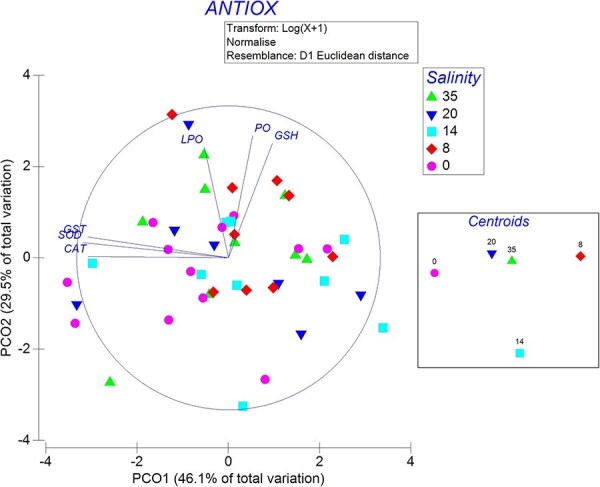
PCO showing the effect of salinity on the antioxidant (ANTIOX) system enzymes—CAT, total glutathione (GSH), GST and SOD—and oxidative stress indicators (LPO and PO) in adult *C. anops*. The right panel displays centroids (central tendency) for each salinity group

**Figure 9:**
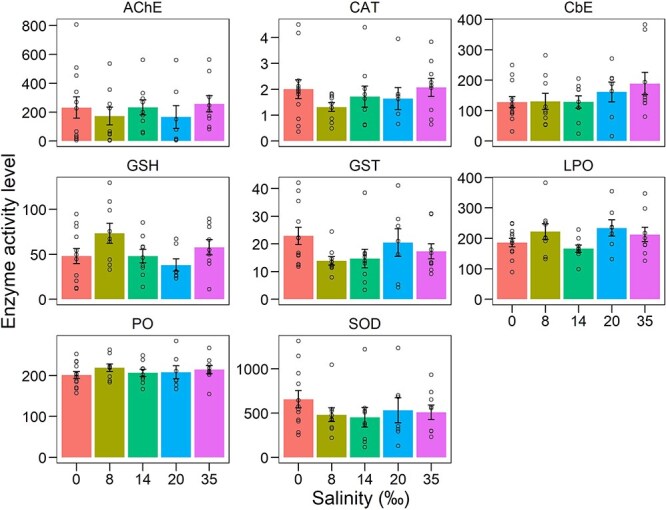
Barplots (mean ± standard error) showing the effect of salinity on antioxidant enzymes (SOD, GSH, GST) and oxidative damage markers (LPO, PO) in adult *C. anops*. Individual points represent replicate values

PERMANOVA results indicated that there was no significant effect of salinity on the ANTIOX system of isopods (Pseudo-*F*_5,6–11_ = 1.12, *P* > 0.05, Unique permutations = 999). The PCO plot (Fig. 8) shows the non-significant response of ANTIOX system enzymes (SOD, CAT, GST and GSH) and oxidative damage markers (LPO and PO) at their native and experimental salinities. PCO1 accounts for 46.1%, whereas PCO2 for 29.5% of the variation in the data, together explaining 75.6% of the total variation. Additional boxplots are provided to aid data interpretation of indicators (Fig. 9).

Despite there were no significant differences among groups, some markers of the ANTIOX system showed interesting trends. Vectors representing the different ANTIOX markers analyzed are categorized into two groups based on their orientation in the PCO plot. One group, consisting of CAT, SOD and GST enzymes, which are involved in primary antioxidative defence, aligns horizontally along the PCO1 axis. The second group, including LPO and PO markers of oxidative damage, along with GSH involved in secondary antioxidative defence, extends along the PCO2 axis.

The PCO plot grouped samples from the control group (0‰ salinity) towards the centre-left side of the ordination, suggesting higher levels of SOD, CAT and GSH activity compared to animals exposed to increasing salinities. Among salinity-exposed animals, those subjected to 8‰ salinity had the lowest enzyme-activities as they grouped towards the right of the plot. Interestingly, all samples exhibit similar levels of LPO, PO and GSH, as no pattern is observed along the PCO2 axis, except for the 35‰ group, which is observed on the upper side of the ordination.

## Discussion

### Characterization of physiological capacities

Our findings suggest that *C. anops* has proficient osmoregulatory capabilities. In the atyid euryhaline shrimp *Halocaridina rubra* of freshwater origin, endemic of the Hawaiian anchialine pools, an isosmotic point close to marine salinity (31‰; ~1000 mOsm/kg H_2_O) was reported, with robust hyper- and hypo-osmoregulation capabilities when challenged with salinity variations ranging from 0 to 56‰ (Havird et al., 2014a). Similarly, *Typhlatya dzilamensis* from the subterranean anchialine environments of the YP, showed remarkable osmoregulation capacities, as they endure acute salinity changes as they traverse through the haloclines from the marine groundwater to fresh groundwater (Chávez Solís et al., 2024). Unlike other typical euryhaline species, such as the marine shrimp *Litopeneus vannamei*, that activate osmoregulation mechanisms “on demand” to cope with salinity changes (Cuenca et al., 2021), *H. rubra* and *T. dzilamensis* appear to maintain constantly active osmoregulatory mechanisms to cope with frequently fluctuating salinities in the anchialine environment (Chávez Solís et al., 2024; Havird et al., 2014a). Similarly to *L. vannamei*, the isosmotic point of *C. anops* (17.7‰), located midway between freshwater and marine water osmotic pressures, supports its capability to colonize both freshwater and saline environments within the Yucatan KSE. Although salinity did not affect significantly oxygen consumption rates, these tended to increase at salinities deviating from the isosmotic point, suggesting a minimal osmoregulation cost.

In addition to its osmoregulatory proficiency, *C. anops* exhibited a thermal tolerance well beyond the temperature changes of its habitat. Thermal tolerance in ectotherms is often closely aligned with climate characteristics, particularly environmental variability (Stevens, 1989), as it governs physiological performance in traits such as growth, survival, and reproduction within a narrower thermal window (Huey, 1991; Mammola et al., 2019; Mermillod-Blondin et al., 2013). Temperatures are notably stable within the KSE, ranging from 23 to 29°C across the YP’s aquifer (Schmitter-Soto et al., 2002; Bauer-Gottwein et al., 2011), with seasonal changes only enhanced after extreme natural phenomena (Kovacs et al., 2017; Brankovits et al., 2022). Despite this stability, *C. anops* demonstrated a thermal tolerance range (CT_max_—CT_min_) of 14.6°C via CT dynamic assays, a range nearly 3-fold greater than the natural thermal variation in its habitat. Notably, while acute temperature tolerance tests (e.g. ramping rates of 1°C/min) may overestimate functional thermal limits under natural conditions (Desforges et al., 2023), this approach allows a standardized comparative protocol with other ectotherms (Rezende et al., 2011; Terblanche et al., 2011; Mermillod-Blondin et al., 2013).

This finding supports emerging evidence that some subterranean species, contrary to assumptions of strict stenothermality, retain relatively broad thermal tolerance ranges (Mermillod-Blondin et al., 2013; Colado et al., 2022; Di Cicco et al., 2023). Comparative studies highlight this variability among groundwater taxa. For instance, two *Proasellus* isopod species exhibited extreme thermal sensitivity (±2°C) under static CT methods, while a third species tolerated an 11°C range, correlating with its broader geographic distribution (Mermillod-Blondin et al., 2013). Similarly, the amphipod *Niphargus inopinatus* displayed a wide 11.7°C thermal preference window, although with signs of stress (e.g. HSP70 upregulation) (Colson-proch et al., 2010; Brielmann et al., 2011). Notably, *C. anops* shares its thermal competence with the co-occurring cave shrimp *Typhlatya* in the YP, which exhibit CT ranges of 20.8–22.2°C (Chávez-Solís et al., 2022). Such parallels suggest that broad thermal tolerance may facilitate colonization of heterogeneous niches in expansive aquifer systems.

While our acute thermal assays revealed a broad thermal tolerance in *C. anops*, long-term metabolic function in ectotherms can be compromised at temperatures well below these critical limits, especially under multifactorial stress (Sokolova et al., 2012; Castaño-Sánchez et al., 2020; Colado et al., 2022). This underscores the necessity of complementing thermal breadth data with insights into bioenergetic mechanisms, such as baseline metabolic rates and biochemical stress markers. This is particularly relevant for *C. anops*, which inhabits sediment microhabitats prone to hypoxia, a factor that may further modulate its physiological responses. Indeed, laboratory experiments to establish baseline metabolic rates showed that *C. anops* had an average routine metabolic rate of 0.024 ± 0.01 mg O₂/g/h. Compared to *Typhlatya* shrimps, which have a routine metabolic rate of 0.19 to 0.33 mg O₂/g/h (Chávez-Solís et al., 2022), *C. anops* exhibited a rate about 10 times lower. This difference may be due to the behavioural traits of *C. anops*, which lives in the sediments, hence are frequently exposed to hypoxia.

Metabolic rates of organisms typically scale with body mass, yet some groundwater species may deviate from this general rule. For most endo- and ectothermic organisms, from unicellular microbes to multicellular plants and animals, metabolic rates typically scale with body mass with an exponent (*b*) ranging from 0.66 to 0.75 (i.e. allometric scaling; e.g. Gillooly et al., 2001; Brown et al., 2004). It has been demonstrated that the surface-to-volume ratio, which scales with a slope of 0.67, has profound effects on metabolic rates, most of them involving changes in the distribution of energy throughout the body (White and Kearney, 2012).

Our data on *C. anops* revealed a slope of 0.70 (*R*^2^ = 0.86), aligning with the recently reported common trend for stygobitic species (Di Lorenzo et al., 2024). The meta-analysis by Di Lorenzo et al. (2024), which analyzed standardized oxygen consumption rate data from 23 studies, found that stygobitic species exhibit an allometric scaling of oxygen consumption rates with a slope of 0.8, suggesting that stygobitic species generally adhere to the scaling patterns observed in terrestrial species. However, growing evidence suggests that some stygobitic species may shift dramatically from this trend. For example, the metabolic rates of adults of the stygobitic amphipod *Gammarus acherondytes* and the stygobitic copepod *Diacyclops belgicus* do not vary with body size (i.e. *b* = 0), unlike most surface water invertebrates (Wilhelm et al., 2006; Di Lorenzo et al., 2015). A similar pattern was also observed for stygobitic isopod *Proasellus lusitanicus* even at different temperatures (Di Lorenzo and Reboleira, 2022). This constant low metabolism, referred to as “ametric” scaling (Di Lorenzo et al., 2015), has been suggested as a physiological adaptation to food-limited environments. Notably, this pattern may only apply to adult stygobites, as isometric scaling has been reported for juveniles of *D. belgicus* (*b* = 1.01; Di Lorenzo et al., 2015). Isometric scaling during development is thought to be a response to the rapid growth rates necessary to avoid predation and reduce juvenile mortality (Glazier, 2006). The size range of the *C. anops* analyzed was 30–390 mg, a 13-fold difference in magnitude. Although published data on the weight range for this species are lacking, the analyzed isopods spanned nearly their entire life cycle, excluding only the earliest juvenile stages. This observation supports the idea that similar environmental pressures inherent to groundwater ecosystems shape the metabolic rates of these organisms. Since excluding outliers modified our scaling coefficient, further research is needed to determine whether the scaling pattern differs for juveniles.

Closed respirometry studies on Portuguese isopod *P. lusitanicus* reported routine metabolic rates of 0.085 mg O₂/g/h at optimal temperatures, which align with values for the *Proasellus* genus (Di Lorenzo and Reboleira, 2022) but are higher than those observed in *C. anops*. One possible reason for the differences observed may be due to the method of analysis. Intermittent respirometry allows for a greater number of measurements, as compared to closed respirometry, and allows the identification of changes in metabolic rates over time. If we consider the early hours of our measurements, *C. anops* showed a relatively higher oxygen consumption rate, yet it was reduced after a few hours. Our findings confirm that the metabolic rates of *C. anops* are within the low characteristic range of other stygobitic crustaceans; however, additional studies using intermittent respirometry are needed to improve comparability and accuracy.

### Responses under stress conditions

*C. anops* showed a strong tolerance to changes in salinity, but this tolerance came with metabolic costs when combined with other stress factors. Initially, we observed a significant increase in routine oxygen consumption rates when exposed to various salinity levels (0, 8, 14 and 35‰). However, after three hours of exposure, there were no significant differences in oxygen consumption rates. This initial increase may be linked to the heightened metabolic demands of osmoregulation (Sokolova et al., 2012), which could later be compensated for, allowing the rates to return to routine levels, thus suggesting their effective compensatory capacity.

However, one might wonder how *C. anops* can be reported to cross the halocline and even inhabit saline waters despite these metabolic costs. We suggest this ability provides an advantage by allowing brief exploration of saline areas to access other resources below the haloclines. There is evidence suggesting that salinity typically has mild effects on the oxygen demand and bioenergetics of aquatic invertebrates within the environmentally relevant range of salinities (Saccò et al., 2022), implying low energy costs of osmoregulation in fully acclimated organisms, which supports our findings.

Interestingly, we could not identify the Pcrit at any of the evaluated oxygen concentrations. The Pcrit represents the threshold of oxygen concentration at which an organism’s aerobic metabolism becomes dependent on oxygen concentration, serving as an index of an organism’s tolerance to ambient hypoxia (Lignot et al., 2000). This result indicates that the isopods could maintain their metabolic rate even at oxygen concentrations as low as 0.5–1 mg/L (~10% saturation), irrespective of salinity. It is well established that hypogean animals can thrive under remarkably low DO levels (Di Lorenzo et al., 2023). However, limited studies have identified the Pcrit in these highly specialized animals (Bishop and Iliffe, 2009). Future work should focus on different times of exposure, as these capacities must bear other physiological costs. The stygobiont amphipods *Niphargus rhenorhodanensis* and *Niphargus virei*, along with the isopod *Stenasellus virei* showed extraordinarily high hypoxia tolerance, with survival times of about two to three days under anoxia and up to several months in moderate hypoxia (Hervant et al., 1996, 1997). The *C. anops* isopods have been observed to inhabit burrows and live under rocks (EC pers. obs.), behaviours that may expose it to local hypoxic environments with extremely low oxygen concentrations, indicating strong adaptive hypoxia tolerance.

Many studies have found that stygobionts have lower metabolic rates than their surface-dwelling counterparts (Hervant et al., 1999; Bishop and Iliffe, 2012; Di Lorenzo and Reboleira, 2022). This metabolic reduction helps them cope with limited energy resources and reduced oxygen availability in groundwater environments. Guo et al. (2018) studied the adaptive evolution of anchialine shrimp of the *Typhlatya-Stygiocaris-Typhlopatsa* complex through 13 mitochondrial genes. Their results suggested a functional convergence of 11 candidate genes towards improved energy metabolism efficiency and enhanced capacity for oxygen utilization. It suggests a positive selection towards an efficient energy metabolism in response to hypoxic cave environments. Tolerance to hypoxia appears to be a necessary adaptation for colonizing groundwater environments, therefore, we hypothesize that *C. anops* should have its own suite of adaptations to such hypoxic environments. Additionally, stygofauna may rely on anaerobic metabolic pathways to generate energy (Hervant et al., 1996, 1997; Havird et al., 2014b; Chávez-Solís et al., 2022). Anaerobic metabolism allows these organisms to continue functioning in conditions where oxygen is scarce, albeit at reduced efficiency compared to aerobic metabolism. All this suggests that, in terms of the adaptation of isopods, their determining factor is likely not DO because they are probably adapted to live in conditions of moderate hypoxia. However, salinity and temperature could play a greater role in the distribution of *C. anops* along the Yucatan aquifer system.

In fact, the antioxidant system profiles suggest mild effects of acute salinity exposure. Animals exposed for just 8 minutes to salinities of 8, 14, 20 and 35‰ showed a reduction in the enzymes SOD, CAT and GST. SOD and CAT are two pivotal enzymes involved in antioxidant defence systems in living organisms (Regoli and Giuliani, 2014). SOD catalyzes the dismutation of superoxide radicals (O_2_^−^) into molecular oxygen (O_2_) and hydrogen peroxide (H_2_O_2_), whereas CAT catalyzes the decomposition of hydrogen peroxide (H_2_O_2_) into water (H_2_O) and molecular oxygen (O_2_), thus playing a primary role as the first defence barrier to neutralize reactive oxygen species (Di Giulio et al., 1989). The reduced activity of these enzymes relative to the control indicates that the increase in oxygen consumption upon acute salinity exposure triggered higher ROS production, leading to a consumption of available enzymes. It was interesting to note that oxidative damage could only be detected at the highest salinity trials (35‰), as revealed by the increase in LPO and PO values. However, the activation of GSH in this group could have contributed to the repair of oxidative damage, as this enzyme participates in the neutralization of lipid radicals and lipid peroxyl radicals, initiated by a lipid-soluble radical scavenger like α-tocopherol (Regoli and Giuliani, 2014). Presumably, the resultant oxidative stress represents minor damage, and its repair mechanisms could be rapid and efficient, as no differences in routine oxygen consumption were observed after three hours. Similar results were found in *Typhlatya*, in which the stimulation of the antioxidant system response in all three *Typhlatya* species accompanied the levels of aerobic metabolism following physical activity (Chávez-Solís et al., 2022; Chávez Solís et al., 2024). Interestingly, the large amount of GSH observed in *T. dzilamensis* may be indicative of an adaptive trait to a more heterogeneous environment. Thus, indicating that this enzyme could also play a role in environmental tolerance in *C. anops*.

## Conclusions

*C. anops* has shown strong compensatory abilities in terms of its tolerance to ambient hypoxia and salinity alterations. This capacity could partially explain its abundance throughout the aquifer system of the Yucatán Peninsula. As a key organism in recycling decomposing matter within the KSE, its presence supports higher trophic level communities across the region.

Although a wide thermal range is observed, its physiological viability is yet to be further evaluated, thus it could be vulnerable to future fluctuations in groundwater temperatures. While groundwater tends to maintain relatively stable temperatures compared to surface water, new evidence suggests that human activities such as deforestation, land use change and urbanization may generate greater impacts than those posed by global climate change, potentially altering temperatures by 2 to 7°C within aquifers (Benz et al., 2024). These issues are also relevant to the Yucatán Peninsula and could jeopardize one of the most important species in the trophic chain. Therefore, it is crucial to generate more information on the thermal biology of the species, including the description of its thermal tolerance limits and adaptive mechanisms.

Changes in salinity could also determine shifts in the distribution of this species, as it showed some sensitivity to salinity. Further information is needed on the capacity of *C. anops* to tolerate different salinities in entirely anoxic environments for longer periods of time.

The Yucatan Peninsula is under significant pressure due to pollution, agriculture, land use change, urbanization and global climate change (Danielopol et al., 2003; Bauer-Gottwein et al., 2011). Continued research efforts focused on Yucatan’s groundwater fauna are essential for unravelling the unique adaptations of stygofauna in this region and advancing conservation efforts to protect these valuable and overlooked ecosystems.

## Data Availability

Data sets are available at Zenodo repository https://doi.org/10.5281/zenodo.14188767.
